# Revisiting Host Preference in the *Mycobacterium tuberculosis* Complex: Experimental Infection Shows *M. tuberculosis* H37Rv to Be Avirulent in Cattle

**DOI:** 10.1371/journal.pone.0008527

**Published:** 2010-01-01

**Authors:** Adam O. Whelan, Michael Coad, Paul J. Cockle, Glyn Hewinson, Martin Vordermeier, Stephen V. Gordon

**Affiliations:** 1 TB Research Group, Veterinary Laboratories Agency Weybridge, New Haw, United Kingdom; 2 School of Agriculture, Food Science and Veterinary Medicine, University College Dublin, Dublin, Ireland; 3 School of Medicine and Medical Science, University College Dublin, Dublin, Ireland; 4 School of Biomolecular and Biomedical Science, College of Life Sciences, University College Dublin, Dublin, Ireland; 5 Conway Institute of Biomolecular and Biomedical Research, University College Dublin, Dublin, Ireland; University of Hyderabad, India

## Abstract

Experiments in the late 19th century sought to define the host specificity of the causative agents of tuberculosis in mammals. *Mycobacterium tuberculosis*, the human tubercle bacillus, was independently shown by Smith, Koch, and von Behring to be avirulent in cattle. This finding was erroneously used by Koch to argue the converse, namely that *Mycobacterium bovis*, the agent of bovine tuberculosis, was avirulent for man, a view that was subsequently discredited. However, reports in the literature of *M. tuberculosis* isolation from cattle with tuberculoid lesions suggests that the virulence of *M. tuberculosis* for cattle needs to be readdressed. We used an experimental bovine infection model to test the virulence of well-characterized strains of *M. tuberculosis* and *M. bovis* in cattle, choosing the genome-sequenced strains *M. tuberculosis* H37Rv and *M. bovis* 2122/97. Cattle were infected with approximately 10^6^ CFU of *M. tuberculosis* H37Rv or *M. bovis* 2122/97, and sacrificed 17 weeks post-infection. IFN-γ and tuberculin skin tests indicated that both *M. bovis* 2122 and *M. tuberculosis* H37Rv were equally infective and triggered strong cell-mediated immune responses, albeit with some indication of differential antigen-specific responses. Postmortem examination revealed that while *M. bovis* 2122/97–infected animals all showed clear pathology indicative of bovine tuberculosis, the *M. tuberculosis*–infected animals showed no pathology. Culturing of infected tissues revealed that *M. tuberculosis* was able to persist in the majority of animals, albeit at relatively low bacillary loads. In revisiting the early work on host preference across the *M. tuberculosis* complex, we have shown *M. tuberculosis* H37Rv is avirulent for cattle, and propose that the immune status of the animal, or genotype of the infecting bacillus, may have significant bearing on the virulence of a strain for cattle. This work will serve as a baseline for future studies into the genetic basis of host preference, and in particular the molecular basis of virulence in *M. bovis*.

## Introduction

Genome sequencing across the *Mycobacterium tuberculosis* complex has underlined the remarkable genetic identity shown between the constituent members, with for example *Mycobacterium bovis* and *Mycobacterium tuberculosis* sharing greater than 99.95% identity at the nucleotide level [1], [2]. However this genetic identity contrasts with the distinct host preference of the tubercle bacilli, which can be seen as series of ecotypes marked by fixed molecular differences [3]. This is evident from epidemiological data, with *M. tuberculosis* a highly successful pathogen of humans, yet not able to sustain in animal populations; the converse is true for *M. bovis*, a pathogen of wild and domesticated mammals that rarely transmits between immunocompetent humans. As the genome sequences of *M. bovis* and *M. tuberculosis* are available, we should now be able to define the genetic differences that lead to host tropism, and therefore to define virulence factors that drive host preference across the tubercle bacilli.

Theobald Smith was the first to describe differences between human and bovine tubercle bacilli [4]. He described detailed characterisation of two bacilli, one an isolate from a pet coati whose owner had died from tuberculosis and which was taken as a human isolate, the other an isolate from a tuberculous bull. Inoculations of these bacilli into guinea pigs, rabbits and cattle showed that the bovine culture was of greater virulence in the animals tested, in particular cattle. Emil von Behring extended this work, producing an attenuated *M. tuberculosis* strain by repeated laboratory culture, and using this “bovo-vaccine” to vaccinate cattle [5]. Robert Koch also investigated the virulence of *M. tuberculosis* for cattle, describing in his 1901 address to the British Congress of Tuberculosis [6] experimental infections of cattle where human “tubercle bacilli or the sputum were injected under the skin, in others into the peritoneal cavity, in others into the jugular vein. Six animals were fed with tuberculous sputum almost daily for seven or eight months; four repeatedly inhaled great quantities of bacilli.” The results of these infections were that “none of these cattle (there were nineteen of them) showed any symptoms of disease and they gained considerably in weight. From six to eight months after the beginning of the experiments they were killed. In their internal organs not a trace of tuberculosis was found” [6]. Hence early work on strain phenotypes indicated that *M. tuberculosis* was avirulent in cattle.

However, it should be noted that the early virulence studies with *M. tuberculosis* and *M. bovis* were limited by the techniques of the day; e.g. strains of *M. tuberculosis* or *M. bovis* were not controlled across laboratories; numbers of bacilli inoculated into experimental animals were not determined; growth conditions and growth phase of the inoculum were ill-defined; and immunological read-outs of infection were non existent. The need for rigorous experimental assessment of the virulence of *M. tuberculosis* in cattle is highlighted by our own isolation of *M. tuberculosis* from cattle in Nigeria and Ethiopia [7], [8] and by the work of others [9], [10], [11], suggesting that the immune status of the animal, or genotype of the infecting bacillus, may have significant bearing on the “virulence” of a strain.

In light of the completion of the genome sequences of *M. tuberculosis* and *M. bovis* and the experimental advances since the seminal studies of Smith, Koch and von Behring, it is now opportune to revisit their early studies of human and tubercle bacilli using the genome sequenced strains of *M. bovis* and *M. tuberculosis*. In this report we describe the experimental infection of cattle with the genome sequenced strains *M. tuberculosis* H37Rv and *M. bovis* AF2122/97, and describe pathological and immunological differences in cattle infected with these strains. The differences between the virulence of these hallmark members of the *M. tuberculosis* complex for cattle will serve as a baseline for future studies into the genetic basis of host preference, and in particular the molecular basis of virulence in *M. bovis*.

## Material and Methods

### Cattle

Approximately 6-month-old calves (Friesian or Friesian crosses, castrated males) were obtained from bovine TB-free herds and were accommodated in a high-level bio-secure facility at VLA. Pre-selection IFN-g assays confirmed their disease-free status. All cattle experiments were cleared by local ethical review and animals procedures performed under a UK Home Office project license within the conditions of the Animals (Scientific Procedures) Act 1986.

### Bacterial Strains

The *M. bovis* field isolate AF2122/97 was grown to mid-log phase in Middlebrook 7H9 media (Difco, UK) supplemented with 10% (v/v) Middlebrook acid-albumin-dextrose-catalase enrichment (Difco), 4.16 g/l sodium pyruvate (Sigma-Aldrich, UK) and 0.05% (v/v) Tween 80 (Sigma-Aldrich) and stocks stored frozen at −80°C. Stocks of *M. tuberculosis* H37Rv were prepared in the same way except that the 7H9 media was supplement with 0.2% (v/v) glycerol (Sigma-Aldrich) rather than sodium pyruvate. The colony forming units (CFU)/ml of bacterial stocks was pre-determined by bacterial enumeration of a serial dilution cultured on modified Middlebrook 7H11 agar [12]. The virulence of both *M. bovis* AF2122/97 and *M. tuberculosis* H37Rv stocks was confirmed via inoculation of guinea pigs prior to the start of the cattle challenge experiment, with both stocks clearly virulent in this model (data not shown).

### Experimental Schedule

Two groups of 5 calves were infected with either *M. bovis* (AF 2122/97) or *M. tuberculosis* (H37Rv) by endobronchial instillation of 1.0×10^6^ and 2.8×10^6^ CFU respectively, as described previously [13], [14]. Blood samples were collected at regular intervals throughout the challenge period. Blood sampling for one of the *M. bovis* infected animals (2757) was only available up until week 5 post-infection since deterioration in the physical condition of this animal was noted and it was euthanazed prematurely at week 6 on animal welfare grounds. Post mortem examination, performed in accordance with the protocol described below, identified extensive miliary TB in this animal. The remaining animals were skin tested with the single intradermal comparative cervical tuberculin test 16 weeks after *M. bovis* infection. The skin tests were performed as specified in the European Economic Community Directive 80/219EEC, amending directive 64/422/EEC, annex B.

### Post-Mortem Examination

At the end of the experimental period (17 weeks post-infection), calves were euthanazed by intravenous injection of sodium pentabarbitone and a *post mortem* examination was performed. Lungs were examined externally for the occurrence of lesions, followed by slicing of the lung into 0.5- to 1-cm-thick sections that were then individually examined for lesions. In addition, lymph nodes of the head and pulmonary regions were removed. Lymph nodes that were subject to detailed analysis always included the submandibular, parotid, medial and lateral retropharyngeal, tonsil, bronchial, cranial and caudal mediastinal and cranial tracheobronchial lymph nodes. All major body organs were also examined for evidence of disease. Lymph node tissues were sliced into thin sections (1 to 2 mm thick) and examined for the presence of visible lesions. Samples were collected for *M. bovis* culture (see below) and also for histopathological examination (Ziehl-Neelsen to stain for acid-fast bacilli and hematoxylin and eosin staining). The severity of the gross pathological changes was scored using a semi-quantitative scoring system as previously described [15]. In summary, each of 7 lung lobes was scored from 0–5 depending on the number of lesions and extent of pathology observed, 0 being no pathology and 5 being extensive gross-coalescing lesions. The lymph nodes of the upper and lower respiratory tract were similarly scored but using a score of 0–3. The scores of the individual lung lobes were added to calculate the lung score and of the lymph nodes to calculate the lymph node score. Both lymph node and pathology scores were combined to determine the total pathology score per animal.

### Bacterial Enumeration

Tissue sections collected at post mortem from lymph node and lung samples were individually homogenized in 5 ml of sterile phosphate buffered saline using a rotating-blade macerator system. Enumeration of CFU/sample was performed by inoculating modified 7H11 agar [12] plates with 50 µl of tissue homogenate and counting colonies after incubation at 37°C for 4–6 weeks. A semi-quantitative bacterial burden score of 0–4 was determined for each tissue sample where 0 = no colonies, 1 = 1–10 CFU, 2 = 11–100 CFU, 3 = 101–1000 CFU and 4>1000 CFU per sample. The score for all tissues was combined to provide a total bacteriology burden score per animal. All culture positive samples from the *M. tuberculosis* infected animals, and a representative sample from 4 of the *M. bovis* infected cattle, were submitted to the VLA Molecular Strain Typing Laboratory to confirm strain identity by spoligotyping. Strains were spoligotyped according to standard methods [16] with minor modifications [8]. All culture positive samples isolated from the cattle infected with *M. tuberculosis* had a spoligotype pattern identical to the H37Rv infection strain whilst cultures from animals infected with *M. bovis* only demonstrated a pattern identical to the AF2122/97 strain (data not shown).

### Antigens and Peptides

Bovine (PPD-B) and avian (PPD-A) tuberculins were supplied by the Tuberculin Production Unit at VLA. For the skin-test, PPD-A and PPD-B were used undiluted. PPD-B was used at a concentration of 10 µg/ml in the IFN-γ ELISPOT and TNF-α ELISA assays. A cocktail of 21 overlapping peptides mapping the entire sequence of both ESAT-6 and CFP-10 was used at a concentration of 5 µg/ml per component peptide in the IFN-γ ELISPOT. Responses to Rv3879c were measured by IFN-γ ELISA using a pool of 10 synthetic peptides previously identified to be recognised by *M. bovis* infected cattle [17].

### IFN-γ ELISPOT Assay

Direct enzyme-linked immunospots (ELISPOTs) were enumerated as described previously [18] using a modification a protocol for indirect ELISPOTs [19]. Briefly, ELISPOT plates (Immobilon-P polyvinylidene difluoride membranes; Millipore, UK) were coated overnight at 4°C with the bovine IFN-γ specific monoclonal antibody 2.2.1. Unbound antibody was removed by washing, and the wells were blocked with 10% fetal calf serum in RPMI 1640 medium (Life Technologies, UK). PBMC [2×10^5^/well suspended in RPMI 1640 media supplemented with 5% controlled process serum replacement type 3 (Sigma Aldrich) nonessential amino acids (Sigma Aldrich) 5×10^−5^M 2-mercaptoethanol, 100 U of penicillin/ml, and 100 µg of streptomycin sulfate/ml (Sigma Aldrich)] were then added and cultured at 37°C and 5% CO2 in a humidified incubator for 24 h. Spots were developed with rabbit serum specific for IFN-γ followed by incubation with an alkaline phosphatase-conjugated monoclonal antibody specific for rabbit immunoglobulin G (Sigma Aldrich). The monoclonal antibody 2.2.1 was kindly supplied by D. Godson (VIDO, Saskatoon, Saskatchewan, Canada). The spots were visualized with 5-bromo-4-chloro-3-indolylphosphate–nitroblue tetrazolium substrate (Sigma-Aldrich).

### IFN-γ ELISA

Whole-blood cultures were performed in 96-well plates in 0.2-ml/well aliquots by mixing 0.1 ml of heparinized blood with an equal volume of antigen diluted in RPMI 1640 supplemented medium as described above. The supernatants were harvested after 48 h of culture at 37°C and 5% CO_2_ in a humidified incubator. The bovine IFN-γ concentration was determined using a commercially available kit (BOVIGAM^®^ ELISA, Prionics AG, Switzerland).

### TNF-α ELISA

PBMC (2×10^5^/well suspended in RPMI 1640 supplemented media as described above) were cultured in the presence of antigen for 6 days at 37°C and 5% CO_2_ in a humidified incubator. Culture supernatants were harvested and the concentration of bovine TNF-α determined using a commercially available kit (Endogen, USA).

### Statistical Analysis

Differences in cellular immune responses between *M. bovis* and *M. tuberculosis* infection groups were compared at multiple post-infection sampling points by non-parametric analysis using the 2-way ANOVA test with Bonferroni post-test analysis. Difference in pathological scoring parameters between groups was determined using the Fisher Exact test, and differences between bacterial burden scores and differences in skin-test responses were compared using the non-parametric Mann-Whitney test with Bonferroni post-test analysis. All statistical calculation were performed using Prism 5 analysis software (Graphpad Inc. USA).

## Results

### Immune Responses in Cattle Infected with *M. bovis* or *M. tuberculosis*

**Figure 1:**
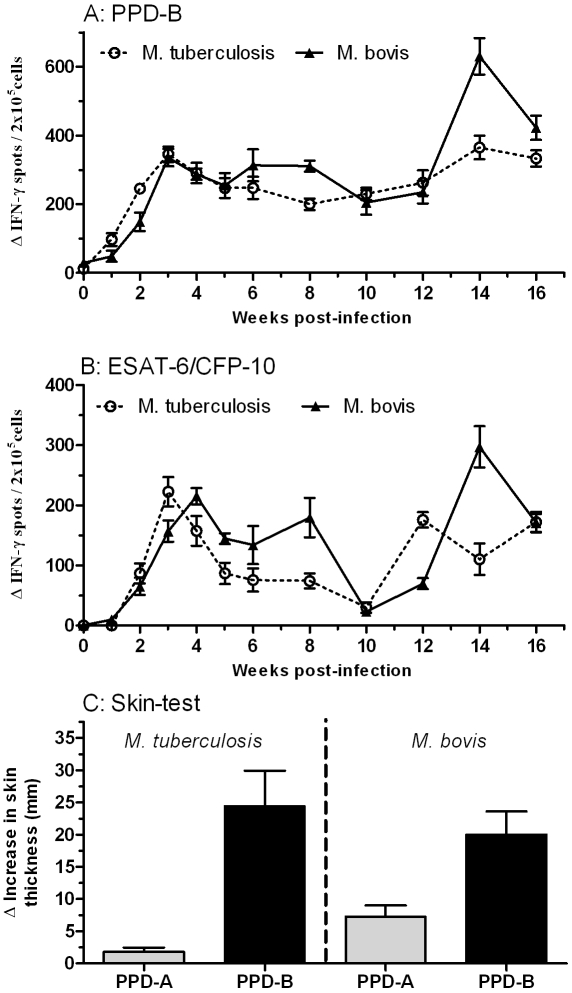
*M. bovis* and *M. tuberculosis* infection induces comparable bovine cellular immune responses. Panels A and B; Blood was collected at regulated intervals from cattle following experimental infection with either *M. bovis* (n = 5) or *M. tuberculosis* (n = 5). PBMCs were isolated and stimulated with bovine-PPD (panel A) or a cocktail of peptides derived from ESAT-6 and CFP-10 (Panel B). The number of antigen-specific cells expressing IFN-γ was measured using an ELISPOT assay. Data for each time point is presented as the mean response ± SEM with the no-antigen response subtracted. The response in the *M. tuberculosis* infected cattle is shown as open circles with a dotted line, and for the *M. bovis* infected cattle as closed triangles with a solid line. Panel C; A tuberculin skin-test was performed at week 16 post-infection. The increase in skin induration was measured 72 hr after administration of bovine (black bars) or avian (grey bars) PPD and is presented as the mean ± SEM.

Infection of cattle via the intratracheal route was achieved using 1.0×10^6^ and 2.8×10^6^ of *M. bovis* and *M. tuberculosis*, respectively. This resulted in specific IFN-γ responses against PPD-B and an ESAT-6/CFP-10 peptide mix developing in all animals starting between one and two weeks post-infection (Figure 1A and B). These responses were maintained throughout the experimental period with no significant differences detectable between animals infected with *M. tuberculosis* or *M. bovis* (Figure 1A and B). Supporting these observations of strong and sustained cellular immune responses induced by *M. bovis* or *M. tuberculosis* infection are the results of the single intradermal comparative cervical tuberculin test shown in Figure 1C. Both infectious agents induced strong bovine PPD biased skin responses (Figure 1C). Interestingly, while we observed stronger skin test responses to avian PPD in the *M. bovis* infected animals compared to *M. tuberculosis* infection (p = 0.0317), the differential between PPD-A and PPD-B responses between groups were not significantly different.

**Figure 2:**
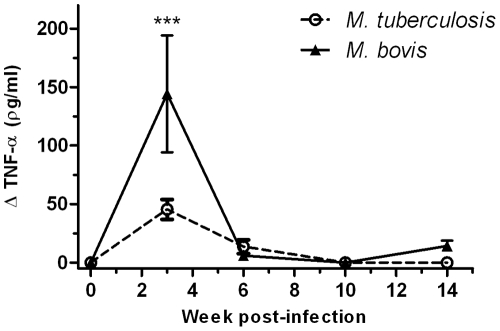
Elevated TNF-α responses in cattle infected with *M. bovis*. Blood was collected from cattle following experimental infection with either *M. bovis* (n = 5) or *M. tuberculosis* (n = 5). PBMCs were isolated, stimulated with bovine-PPD and the expression of TNF-α measured in the culture supernatant by ELISA. The response in the *M. tuberculosis* infected cattle is shown as open circles with a dotted line, and for the *M. bovis* infected cattle as closed triangles with a solid line. Data for each time point is presented as the mean response ± SEM with the no-antigen response subtracted. Significant differences between groups was determined using 2-way ANOVA with Bonferroni post-test analysis (*** p<0.001).

In addition to IFN-γ responses we also measured *in vitro* TNF-α responses after PBMC stimulation with bovine tuberculin. Responses were measured by ELISA and are shown in Figure 2. TNF-α production peaked 3 weeks post-infection and subsequently waned to background levels in both groups. However, the responses of *M. bovis* infected animals were at this response peak time point significantly higher compared to *M. tuberculosis* infected animals (P<0.001, Figure 2).

**Figure 3:**
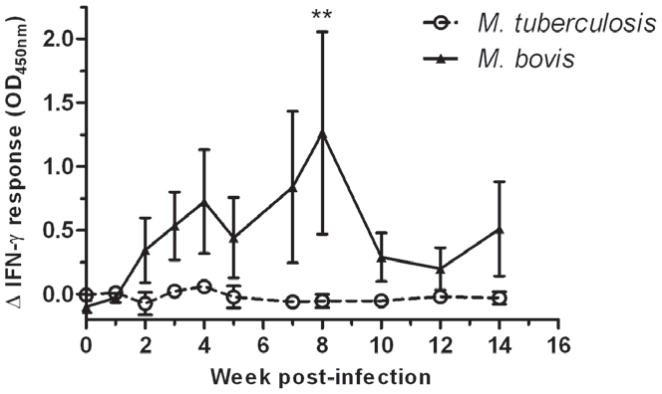
Differential recognition of Rv3879c by *M. bovis* infected cattle. Blood was collected from cattle following experimental infection with either *M. bovis* (n = 5) or *M. tuberculosis* (n = 5). Whole blood was stimulated with a pool of peptides derived from Rv3879c and expression of IFN-γ was measured by ELISA. The response in the *M. tuberculosis* infected cattle is shown as open circles with a dotted line, and for the *M. bovis* infected cattle as closed triangles with a solid line. Data for each time point is presented as the mean response (OD_450nm_) ± SEM with the no-antigen response subtracted. Significant differences between groups was determined using 2-way ANOVA with Bonferroni post-test analysis (** p<0.01).

We further measured *in vitro* IFN-γ responses induced by a number of antigen candidates prioritized as diagnostic reagents (Cockle et al. 2002) including the RD1 antigen Rv3879c. Whilst we could not detect response differences to other antigens tested (as shown for ESAT-6 and CFP-10 in Figure 1B, with data for others not shown), we observed a striking difference in the responses to Rv3879c between *M. bovis* and *M. tuberculosis* infected cattle in that T cells from the *M. tuberculosis* infected cattle did not respond to stimulation with this antigen, whilst their counterparts from *M. bovis* infected calves did (Figure 3). Although this difference was apparent at almost all time-points tested, it reached statistical significance at week 8 post-infection (P<0.01). Thus both the differences in TNF-α production and the differences in T cell specificity induced by the two pathogens suggested a different outcome of infection which was assessed 17 weeks post-infection by post-mortem examination (see next section).

### Pathological and Microbiological Assessment of Virulence

Of the *M. bovis* infected cattle, animals 2735 and 2756 showed mild clinical signs of tuberculosis within the ethically acceptable severity limit, while animal 2757 developed significant clinical disease and had to be euthanazed after 6 weeks due to welfare considerations. Seventeen weeks after infection, all remaining animals were culled and detailed post-mortem examinations were carried out. Pathology was scored using a semi-quantitative method which we previously developed [15]. From the post-mortem examinations, it was clear that while *M. bovis* had caused extensive gross pathology demonstrated by the presence of visible lesions in the lungs and associated lymph nodes, cattle infected with *M. tuberculosis* showed no pathological signs of disease (Table 1). Statistically, these difference were highly significant for the numbers of affected lung lobes and lung-associated lymph nodes, as well as the lymph node, lung, and total pathology scores (P<0.01). The bacterial culture results showed that while all *M. bovis* infected animals were culture positive, only 3/5 cattle infected with *M. tuberculosis* were culture positive (Table 1). In addition, when we applied a culture score to describe bacterial loads in the lymph nodes cultures, we also observed a significantly lower bacterial burden (Table 1, p<0.01). Hence, it appears that while *M. tuberculosis* was able to persist in the majority of animals at relatively low bacillary loads, it was incapable of causing pathology.

**Table 1:** With the exception of animals 2757, post mortems were performed 16 weeks post infection.

Animal^a^	Pathology^b^						Bacteriology^c^
	Lung		Lymph Nodes			Total Pathology	
	Lobes affected**	Score**	*Head LNs*	Lung LNs**	LN Score**	Score**	Score**
***M. tuberculosis***							
2730	0	0	0	0	0	0	1
2731	0	0	0	0	0	0	5
2732	0	0	0	0	0	0	0
2733	0	0	0	0	0	0	6
2734	0	0	0	0	0	0	0
***M. bovis***							
2723	3	11	2	3	14	25	27
2735	4	15	1	5	16	31	16
2736	2	9	0	2	3	12	15
2756	5	20	0	5	15	35	23
2757	7	30	4	5	24	34	55

Post mortem for 2757 was performed at week 6 post infection for reasons of animal welfare. The number of individual lung lobes and respiratory tract associated lymph nodes with TB lesions is presented. The pathology score is based on a semi-quantitative assignment of disease severity within affected tissues as described in the material and methods. Statistical difference between groups was determined using the Fisher Exact test (** p<0.01). Bacteriology score is based on the subtotal of a semi-quantitative ranking of total CFU for each individual tissue homogenate as described in the material and methods. Statistical difference between groups was determined using the Mann-Whitney test (** p<0.01).

## Discussion

It is well acknowledged that *M. tuberculosis* is a human-adapted pathogen with an exquisite ability to infect and persist in human populations. Infection of animals by *M. tuberculosis* has been reported, but again it is generally accepted that this represents spill-over of infection from humans to animals, and that animal populations cannot sustain *M. tuberculosis*. This apparent attenuation of *M. tuberculosis* in animal hosts is all the more intriguing given that genome studies have shown us that the *M. bovis* genome is merely a reduced version of the *M. tuberculosis* genome; hence, *M. bovis* does not have any “virulence” loci for animals *per se* that have been lost in *M. tuberculosis*. Instead it appears likely that differential expression of a range of genes between *M. tuberculosis* and *M. bovis* explains their specific host predilections.

We therefore sought to revisit the virulence of *M. tuberculosis* for bovines using the genome sequenced type strain, *M. tuberculosis* H37Rv, the best characterized strain of *M. tuberculosis* currently available. As a control we used the *M. bovis* AF2122/97 strain, the genome sequenced strain of *M. bovis* that we have used to develop our bovine infection models. From our results it was clear that *M. tuberculosis* H37Rv was avirulent in cattle; no pathological signs of disease were evident in any of the infected cattle. This is despite the fact that this same seed lot used to infect cattle was capable of causing disease in guinea pigs (data not shown). Hence under precise experimental conditions *M. tuberculosis* H37Rv and *M. bovis* AF2122/97 have unambiguous phenotypic differences in cattle. The immunological analysis of IFN-γ and tuberculin skin test responses, though, clearly indicated that both species are equally infective and cause strong cell-mediated immune responses. The reduced production of TNF-α in the *M. tuberculosis* H37Rv infected cows is in agreement with the lack of visible pathology and reduced virulence as TNF-α has been implicated in murine models in formation and maintenance of granulomas (e.g. [20]). It has also not escaped our notice that the model of *M. tuberculosis* infection of cattle could constitute a potential animal model for human latency.

The difference seen in the recognition of Rv3879c between *M. bovis* and *M. tuberculosis* infected animals is puzzling. Transcriptome analysis of *M. bovis* and *M. tuberculosis in vitro* revealed no difference in the expression of Rv3879c between the two strains [21]; studies looking at intramacrophage gene expression of *M. bovis* and *M. tuberculosis* in bovine alveolar macrophages also showed no difference in Rv3879c expression (unpublished). Rv3879c is known to show sequence variation between *M. bovis* and *M. tuberculosis* [22], but the immunodominant epitopes do not map to the variable region (data not shown), indicating that this is not the reason for the differential recognition. Further investigation of the differential recognition of antigens by *M. tuberculosis* and *M. bovis* may shed light on the biological basis for this observation.

Isolation of *M. tuberculosis* from cattle in countries that include India, Nigeria, Ethiopia and China [7], [8], [9], [11] may point not only to the weight of *M. tuberculosis* infection in these countries, but also to the husbandry conditions and immune status of the cattle. It appears therefore that distinct *M. tuberculosis* genotypes, or the immune status of the animals, or a combination of these factors, plays a major part in the outcome of infection of cattle with *M. tuberculosis*.

The genetic basis for some of the key traits that differentiate *M. bovis* from *M. tuberculosis* have now been deciphered, meaning that we can construct recombinants where *M. tuberculosis* expresses traits normally associated with *M. bovis*. Attractive candidates are the antigens Mpb70 and Mpb83, proteins that are expressed at much greater levels in *M. bovis* than in *M. tuberculosis* [23]. Behr and colleagues have shown that these antigens are controlled by the SigK regulon, and that *M. bovis* has a defective copy of the negative regulator, the anti-SigK, leading to overexpression of these antigens in *M. bovis* [24]. Furthermore, the Oryx bacillus shows an independent mutation leading to SigK regulon dysregulation, suggesting that overexpression of this particular regulon may confer some selective advantage for infection of animals [24]. Another factor that may confer a selective advantage on *M. bovis* is mycoside B, a monosaccharide phenolic glycolipid (PGL) produced by all strains of *M. bovis* but not by *M. tuberculosis* [25], [26]. It has previously been shown that the related, trisaccharide PGL produced by Beijing strains of *M. tuberculosis* can act as an immunomodulator [27]. Hence mycoside B may act to manipulate the bovine immune response to *M. bovis* infection.

In conclusion, we have revisited some of the classic early work on the infection of cattle with *M. tuberculosis* using an experimental infection model and genome sequenced strains of *M. tuberculosis* and *M. bovis*. This work sets the baseline for a dissection of the virulence factors of *M. bovis*, and in defining a molecular basis for host tropism in the *M. tuberculosis* complex.
